# Systemic Immune Activation and Responses of Irradiation to Different Metastatic Sites Combined With Immunotherapy in Advanced Non-Small Cell Lung Cancer

**DOI:** 10.3389/fimmu.2021.803247

**Published:** 2021-12-14

**Authors:** Min Wu, Jie Liu, Shihao Wu, Jingru Liu, Hui Wu, Jinming Yu, Xue Meng

**Affiliations:** ^1^ Cheeloo College of Medicine, Shandong University, Jinan, China; ^2^ Department of Radiation Oncology, Shandong Cancer Hospital and Institute, Shandong First Medical University and Shandong Academy of Medical Sciences, Jinan, China; ^3^ Medical School, Anhui University of Science and Technology, Huainan, China; ^4^ Department of Radiation Oncology, Affiliated Cancer Hospital of Zhengzhou University, Zhengzhou, China

**Keywords:** NSCLC, radioimmunotherapy, irradiated organs, inflammatory blood indexes, immune activation effect

## Abstract

**Purpose:**

Considering the limited data, we aimed to identify the greatest immune activation irradiated site of common metastases and response to immune checkpoint inhibitors simultaneously in non-small cell lung cancer (NSCLC).

**Methods:**

A total of 136 patients with advanced NSCLC who had received radiation to a primary or metastatic solid tumor were enrolled. We recorded blood cell counts in three time periods, before, during, and after radiotherapy (RT), and derived some blood index ratios including monocyte-to-lymphocyte ratio (MLR), neutrophil-to-lymphocyte ratio (NLR), platelet-to-lymphocyte ratio (PLR), and systemic immune-inflammation index (SII). The delta-IBs were calculated as medio-IBs ÷ pre-IBs − 1. We analyzed the changes before and during RT using Spearman rank correlation test, Kruskal–Wallis rank sum test, and logistic regression analyzing their correlation with efficacy.

**Results:**

The medians of delta-MLR and delta-PLR were both the lowest while the median of delta-L was the highest in brain. Therapeutic effect evaluation showed that the objective response rate (ORR) of 48.65% (18/37) in the brain irradiation group was the highest, compared with 17.07% (7/41) in bone and 41.94% (13/31) in lung.

**Conclusions:**

In this study, results suggested that irradiation to brain has the best immune activation effect and patient outcome compared with other organs in NSCLC, and when the earlier-line ICIs were combined with RT, a better patient outcome was reached. Prospective studies are also necessary to provide more convincing evidence and standards for clinical irradiation metastases selection.

## Introduction

Advanced NSCLC is the most common pathological type in lung cancer with a 5-year survival rate of less than 5% (1). After the great success of targeted therapy, immune checkpoint inhibitors (ICIs) mainly targeting programmed cell death receptor-1 (PD-1)/programmed cell death receptor-ligand 1 (PD-L1) have shown great survival improvement in advanced NSCLC in recent years (2). The facilitation of augmenting immunotherapy includes increasing the release of tumor antigens and T-cell infiltration and enhancing antigen presentation (3). However, as a solution to overcoming immunotherapy resistance, radiotherapy seems more effective (4–6). Current lines of evidence indicate that ionizing irradiation (IR) seems inadequate to maintain antitumor immunity due to common local relapses (3, 7). However, stereotactic body radiation therapy (SBRT) is a novel mode of radiotherapy that achieves local control rate range from 70% to 90% compared with conventionally fractionated radiotherapy (CFRT) especially in early-stage and oligometastatic NSCLC patients (8–10). SBRT combined with ICIs can make mutual significant progresses respectively, achieving the goal of “1+1>2”. However, there is little study related to the specific number and localization of irradiated lesions for advanced NSCLC patients with multiple metastases, which should be considered as stratification factors (11).

RT mainly activates immune system by enhancing antigen presentation, as well as increasing the infiltration of inflammatory cells (3). Studies revealed that in the development and progression of cancer, inflammation is also a major driver including the fighting among neutrophils, macrophages, lymphocytes, and tumor cells in immunotherapy (12–14). In recent years, a variety of clinical studies have shown inflammatory cells and some derived ratios are strongly associated with the prognosis of patients in solid tumors, which is lack of biomarkers except for PD-L1 and tumor mutational burden (TMB) that is not prospectively validated though (15–18). Considering the limitations of a clinical retrospective study in NSCLC, the inflammatory indicators we collected can indirectly reflect the activation of immune system during RT. Studies have reported that once the anti-cancer therapy works, the immune status of patients must be improved by increasing lymphocyte counts and decreasing monocytes, resulting in an increase of lymphocyte counts and LMR (19). Moreover, lower PLR may be associated with a better survival and higher response rates in immunotherapy and negative prognostic value is shown in SII as well, which might help guide therapeutic strategies in immunotherapy (17, 20, 21). When irradiation was involved in combination with immunotherapy, the above inflammatory biomarkers changed characteristically due to the difference radiosensitivity in immune cells (22). Hypofractionated stereotactic radiation therapy (HSRT) can not only increase the frequency of lymphocytes, especially CD8+ T cells, but also decrease inhibitory Tregs, which has an impact on the immune activation and functional properties of T lymphocytes in cancer patients (23, 24).

In terms of lung cancer, common distant metastatic sites include brain, bone, liver, adrenal gland, contralateral lung, and draining lymph node, which indicate a poor outcome frequently (25). However, as the genetic heterogeneity between the primary and metastatic lesions, different irradiated sites differing in inducing immunogenic cell death (ICD) and durable anti-tumor immunity (26). Temporal variability and the inter- and intra-tumoral spatial heterogeneity including mutation profiles and tumor immune microenvironment are also found to be different (27). Studies have revealed the site-specific metastatic timing, indicating that lymph node is susceptible to early metastatic seeding but pleura and some distant sites tend to seed lately (28). Unique genetic alterations are identified for different metastatic locations, which result in altered molecular pathways and protein expressions, responding to immunotherapy, respectively (29, 30). Immunotherapy as well as RT for different metastases can make different immune system changes. A study has suggested that stereotactic ablative radiation therapy (SAR) induced systemic immunologic changes dependent on irradiated sites (31), which indicated that the combination therapy of RT to different organs and immunotherapy probably affects synergistically. The purpose of this study is to figure out the greatest immune activation effect among different irradiated sites during immunotherapy, so as to provide more beneficial clinical options for advanced NSCLC patients.

## Methods

### Patients

This retrospective study intended to find out the strongest irradiated sites in immune activation effect and treatment response with immunotherapy in advanced NSCLC. A total of 136 patients were included in the analysis, who had received radiation to any organ for a primary or metastatic solid tumor during immune monotherapy or combined with chemotherapy or vascular endothelial growth factor receptor (VEGFR) therapy in Shandong Cancer Hospital from July 2018 to February 2021. The inclusion criteria included age, gender, height, weight, smoking, drinking, Karnofsky performance status (KPS), TNM staging, pathological pattern, treatment stage, medication of immunotherapy, and the modality of RT. The exclusion criteria included SCLC patients, age under 18 years old, KPS score under 70, III or earlier-stage NSCLC, and radiotherapy before or after immunotherapy. The patients were all staged according to the American Joint Committee of Cancer eighth edition TNM classification and staging system (32). The data in this retrospective study did not require ethical certification.

### Treatment Characteristics

All patients were treated with ICIs intravenously every 3 weeks regardless of the expression of PD-1 or PD-L1. Some of them were subjected to combination chemotherapy and the regimens included pemetrexed, docetaxel, and gemcitabine, with or without platinum. VEGFR was added to some patients, and several patients were treated with the combination of the above three therapeutic regimen. As for the VEGFR, the representations are mainly Bevacizumab and Anlotinib. During the immunotherapy, patients underwent 10 to 30 fractions of CFRT at 1.7–4.5 Gy per fraction, or 5 to 10 fractions of SBRT at 5.0–10.0 Gy per fraction for cancer of any histologic type and site (including brain, bone, lung, liver or adrenal gland, and other organs) simultaneously. These sites were irradiated according to patients’ symptoms or clinical relevance preferentially, once daily, five fractions per week. Radiation plans were normalized in that 95% of the plan tumor volume received 100% of the prescribed dose. Besides, doses of organs at risk (OARs) were limited within the safe range.

### Blood Index Collection

Blood cell counts were recorded for all patients in three time periods. The first stage was about 1 month before RT, during which the blood indicators were called pre-inflammatory biomarkers (pre-IBs). The other two stages were during and after radiotherapy, called medio-inflammatory biomarkers (medio-IBs) and post-inflammatory biomarkers (post-IBs), respectively. Pre-IBs were specifically defined as from the first day of immunotherapy to the beginning of radiotherapy. Medio-IBs simply referred to the blood indicators between the first and last day of radiotherapy. While considering the memory effect of immunotherapy, post-IBs were defined as the period from the end of radiotherapy to 2 months after the end of immunotherapy, before the next line treatment. We recorded blood cell counts more than once in each period and then averaged or derived them, including neutrophils, lymphocytes, monocytes, platelets, MLR, NLR, PLR, and SII. The delta-IBs were calculated as follows:


delta−IBs=medio−IBs÷pre−IBs–1

### Response Evaluation

The evaluation of the RT to different sites during immunotherapy was based on immune response criteria in solid tumor (iRECIST) (33). The enhanced magnetic resonance imaging (MRI) and computed tomography (CT) of brain, neck, chest, and abdomen of all patients were evaluated by at least two deputy or chief physicians in the Department of Imaging and Radiation, respectively, according to iRECIST. Tumor control included complete response (CR) and partial response (PR), which were classified as objective responders, while stable disease (SD) and progressive disease (PD) were defined as non-responders. Clinical benefit rate (CBR), including SD, PR, and CR, was used considering the limited number of responders.

### Statistical Analysis

We converted the continuous variables in the study into binary variables by using receiver operating characteristic (ROC). Spearman rank correlation test was used to compare the correlation between blood indexes and different irradiated groups and short-term efficacy. If the correlation coefficient is greater than 0.2, it is considered to have statistical correlation. Kruskal–Wallis rank sum test was used to determine which indicators have differences within groups and then to compare pairwise with a corrected *α*, whose significance was assumed at less than 0.0083 (0.05/6). Univariate logistic regression analysis was performed to evaluate the effect of independent variables on short-term efficacy. We also conducted the factors whose *p*-value were lower than 0.05 into multivariate analysis in case of missing indicators that might have clinical significance. IBM SPSS Statistics software, version 25.0 (SPSS Inc., Chicago, IL), was used for statistical analysis.

## Results

### Patient Characteristics

Clinical baseline characteristics of the 136 patients enrolled are summarized in **Table 1**, which were divided into 6 groups according to different irradiated organs, namely, brain, bone, lung with or without drainage area lymph node, liver, adrenal gland(s), and soft tissue. Delta-MLR, delta-NLR, delta-PLR, delta-SII, delta-L, and delta-M of three patients and delta-EOS of eleven patients were lost. Short-term response after radiotherapy was evaluated availably in 128 patients.

**Table 1:** Demographics and patient baseline characteristics.

Characteristics	No. (%)
Sex	
Male	101 (74.3)
Female	35 (25.7)
Age (years)	
≤60	75 (55.0)
>60	61 (45.0)
ECOG performance status	
≤1	124 (91.2)
≥2	12 (9.7)
Smoking status	
Never	67 (49.3)
Current or former	69 (50.7)
Pathological pattern	
Squamous cell carcinomas	35 (25.7)
Adenocarcinoma	91 (66.9)
Others	10 (7.4)
Treatment stage	
First-line	31 (22.8)
Second-line	62 (45.6)
Third-line and more	43 (31.6)
Stage of disease	
IV (M1a)	11 (8.1)
IV (M1b–M1c)	125 (91.9)
Irradiated sites	
Brain	39 (28.7)
Bone	43 (31.6)
Lung (drainage area lymph node)	33 (24.3)
Liver	7 (5.1)
Adrenal grand	10 (7.4)
Soft tissue	4 (2.9)

No., number; ECOG, Eastern Cooperative Oncology Group.

### Irradiated Organs Correlation With Blood Indicators

**Figure 1:**
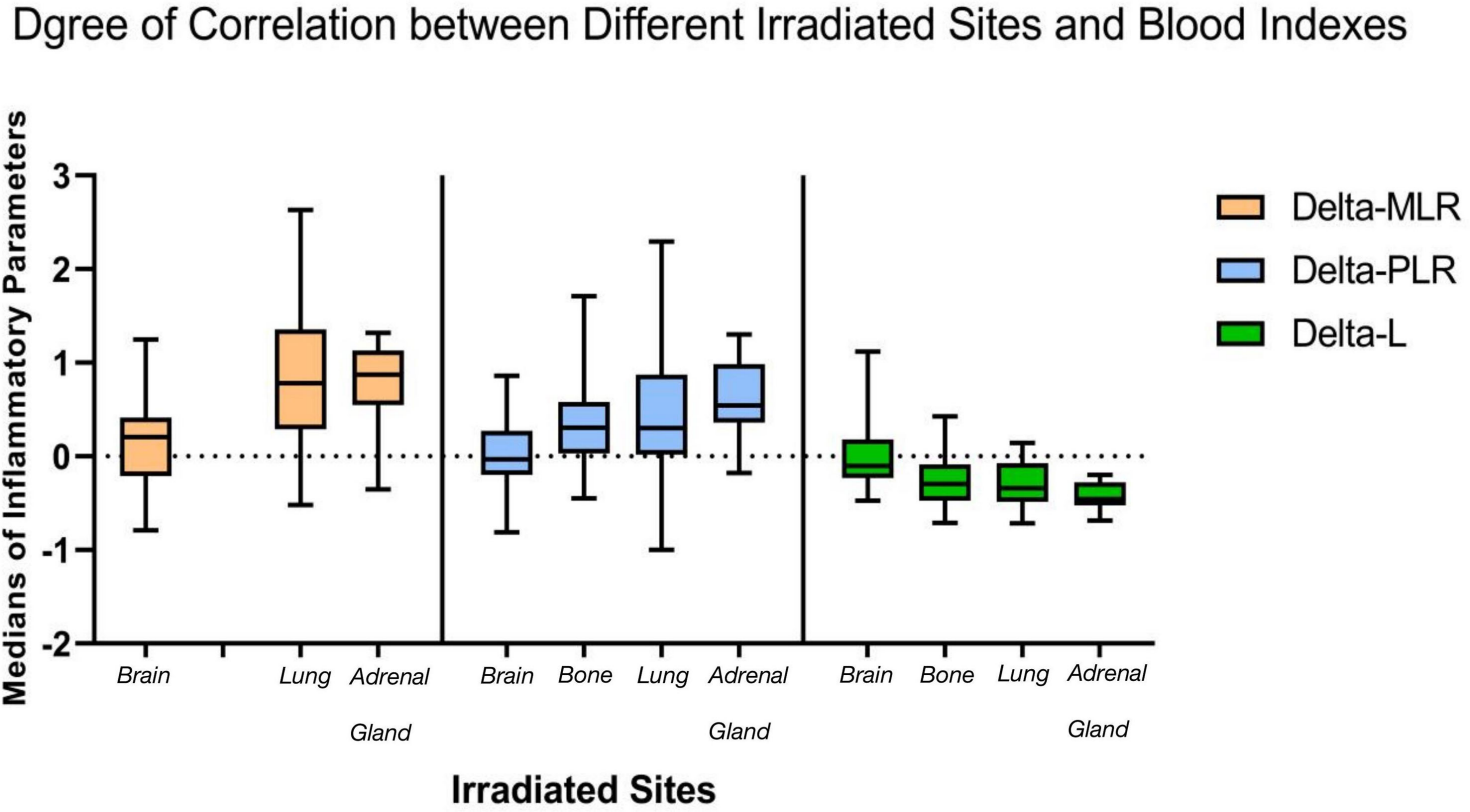
Statistical differences among groups exist in three inflammatory indicators. The medians of delta-MLR and delta-PLR were the lowest and the median of delta-L was the highest in the brain radiation group during immunotherapy, while they were similar in bone and lung. which were both better than adrenal gland. MLR, monocyte-to-lymphocyte ratio; PLR, neutrophil-to-lymphocyte ratio.

Spearman rank correlation test suggested that four of seven delta-IBs have correlation with groups, among which delta-MLR, delta-PLR, and delta-SII were positive (*r* = 0.339, 0.383, and 0.271, *p* < 0.001) and delta-L was negative (*r* = −0.381, *p* < 0.001). After Kruskal–Wallis rank sum test, it was found that three of these four indicators have inter-group differences, namely, delta-MLR, delta-PLR, and delta-L (*p* = 0.001, 0.001, and 0.000). Then, we performed pairwise comparison among groups based on the three indicators, and significance was assumed at *p* less than 0.05 (**Table 2**). We found that the brain irradiation group has statistical difference with the lung and adrenal gland group in delta-MLR (*p* = 0.000 and 0.002). The median was lowest in the brain group, but highest in adrenal gland (medians = 0.208, 0.780, and 0.871). Similarly, in delta-PLR, there was statistical difference between the brain group compared with the bone, lung, and adrenal gland group (*p* = 0.002, 0.004, and 0.000), and the medians of the four groups increased in turn (medians = −0.029, 0.307, 0.302, and 0.543). However, it was the highest in brain in terms of delta-L (medians = −0.104, −0.300, −0.340, and −0.457), which statistically differed from bone, lung, and adrenal radiotherapy groups as well (*p* = 0.001, 0.000, and 0.000). Besides, there were subtle differences between lung and bone in meaningful indicators (**Figure 1**).

**Table 2:** Correlation between blood indexes and RT groups as well as short-term efficacy.

Characteristics	Delta-MLR	Delta-NLR	Delta-PLR	Delta-SII	Delta-L	Delta-M	Delta-EOS
Groups (irradiated sites)	**0.339**	0.175	**0.383**	**0.271**	−**0.381**	−0.041	0.195
Inter-group difference (*p*-value)	0.001		0.001	0.055	0.000		
Short-term efficacy	−0.024	0.017	0.163	0.122	−0.147	−0.138	−0.172

RT, radiotherapy; MLR, monocyte-to-lymphocyte ratio; NLR, neutrophil-to-lymphocyte ratio; PLR, platelet-to-lymphocyte ratio; SII, systemic immune-inflammation index; L, lymphocyte; M, monocyte; EOS, eosinophils.

The bold values mean the correlation coefficients between blood indexes and different irradiated groups in Spearman rank correlation test. Similarly, the "0.000" means positive blood indexes differ in specific irradiated sites via Kruskal-Wallis rank sum test.

### Irradiated Organs Correlation With Immune Response

Among the 128 evaluated patients, no patient reached CR. A total of 41 patients reached PR (18 in brain, 7 in bone, 13 in lung, 1 in liver, and 2 in adrenal gland), 72 patients were SD (15 in brain, 30 in bone, 17 in lung, 4 in liver, 4 in adrenal gland, and 2 in soft tissue), and 15 patients were PD (4 in brain, 4 in bone, 1 in lung, 2 in liver, 2 in adrenal gland, and 2 in soft tissue), yielding an ORR of 32.03% (41/128) and a CBR of 88.28% (113/128).

In the univariate analysis of Binary logistic regression, we found that two factors were significantly associated with short-term efficacy, which were groups [OR, 1.312; 95% CI, 1.041–1.652; *p* = 0.021] and treatment stage [OR, 5.436; 95% CI, 1.955–15.118; *p* = 0.001] (**Table 3**). Besides, no relationship was discovered between all blood indicators and immune response. The two independent variables with *p* < 0.05 in univariate analysis were conducted to multivariate analysis. We found that treatment stage [OR, 4.859; 95% CI, 1.723–13.700; *p* = 0.003] was the only one independent factor associated with response in multivariate analysis. After sub-group univariate analyses according to different irradiated sites, it was found that brain, bone, and lung had statistically significant correlations with short-term efficacy, with *p* < 0.05. As for treatment line, first-line to third-line therapy all statistically associated with therapeutic efficacy (*p* = 0.000, 0.000, and 0.009; OR = 0.077, 0.091, and 0.393).

**Table 3:** Univariate analysis of clinical characteristics and inflammatory parameters in correlation with short-term efficacy.

Parameters	*p*-value	OR	95% CI
**Clinical Characteristics**			
Age (≤60 vs. >60), years	0.351	1.653	0.575–4.752
Gender (Male vs. Female)	0.939	0.954	0.285–3.193
BMI	0.157	0.899	0.777–1.042
Pathological pattern			
Squamous cell carcinomas vs. Adenocarcinoma and Others	0.254	0.551	0.198–1.535
Treatment stage			
First-line vs. Second-line and Third-line and more	0.001	5.436	1.955–15.118
ICIs modalities			
Monotherapy vs. CT combined vs. VEGFR combined and three modes combined	0.349	1.338	0.727–2.464
RT segmentation model (CFRT vs. SBRT)	0.883	1.178	0.132–10.471
Smoking status (Never vs. With)	0.894	0.931	0.327–2.655
Drinking status (Never vs. With)	0.376	0.497	0.106–2.335
ECOG performance status (≤1 vs. ≥2)	0.760	1.257	0.290–5.458
Groups (irradiated sites)	0.021	1.312	1.041–1.652
Brain	0.000	0.121	–
Bone	0.000	0.108	–
Lung (drainage area lymph node)	0.000	0.069	–
Liver	0.273	0.400	–
Adrenal grand	0.178	0.333	–
Soft tissue	1.000	1.000	–
**Inflammatory Parameters**			
Delta-MLR	0.328	2.188	0.456–10.499
Delta-NLR	0.446	0.762	0.379–1.534
Delta-PLR	0.978	0.946	0.019–46.332
Delta-SII	0.735	1.412	0.191–10.406
Delta-L	0.985	1.033	0.031–34.744
Delta-M	0.101	0.083	0.004–1.621
Delta-EOS	0.249	0.698	0.379–1.285

CI, confidence interval; BMI, body mass index; ICIs, immune checkpoint inhibitors; VEGFR, vascular endothelial growth factor receptor; CT, chemotherapy; CFRT, conventional fractionated radiotherapy; SBRT, stereotactic body radiotherapy; ECOG, Eastern Cooperative Oncology Group; MLR, monocyte-to-lymphocyte ratio; NLR, neutrophil-to-lymphocyte ratio; PLR, platelet-to-lymphocyte ratio; SII, systemic immune-inflammation index; L, lymphocyte; M, monocyte; EOS, eosinophils.

**Figure 2:**
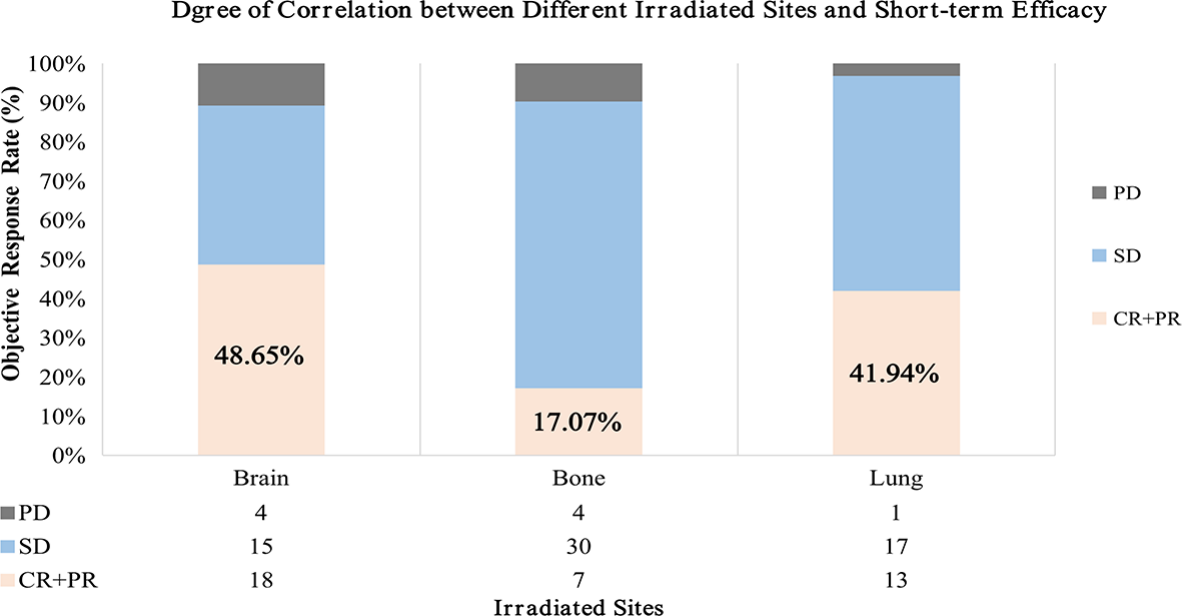
Short-term efficacy differs statistically in three groups. The result showed that the ORR of brain irradiation group was the highest compared with bone and lung. ORR, objective response rate; CR, complete response; PR, partial response; SD, stable disease; PD, progressive disease.

The therapeutic evaluation of each group showed that the ORR of 48.65% (18/37) in the brain irradiation group was the highest, compared with 17.07% (7/41) in the bone group and 41.94% (13/31) in the lung group (**Figure 2**). The CBR reached 92.9%, 91.7%, and 71.8% in the first-, second-, and third-line therapy, respectively.

## Discussion

The combination of radiotherapy and immunotherapy seems to be a major treatment in NSCLC in the future. However, compared with more regulated immunotherapy, the irradiated site and modality of radiotherapy are still inconclusive to date (4). In clinical practice, in addition to palliative RT for patients with obvious symptoms, the intervention of RT for multiple metastases has always been questionable, regardless of the timing, mode, or site (11).

The results of this analysis that enrolled 136 patients showed that the medians of delta-MLR and delta-PLR were the lowest and that of delta-L was the highest in the brain radiation group during immunotherapy. Considering the significance of blood indicators in the immune system (34), we hypothesize that irradiation to brain has the strongest activation effect and anti-tumor response on inflammatory. Moreover, in the subgroup analysis of short-term efficacy in different irradiation sites, it was also found that patients with brain RT had the best disease control, reaching an ORR over 48%. About the reason, we thought that it may be due to the fact that the blood–brain barrier (BBB) was broken with RT. However, the ORR was significantly lower in bone than that in lung, the reason for which might have depended on patients’ subjective feeling instead of the obvious visible lesion reduction radiographically, leading mostly to SD. On the contrary, more PR and CR were reached in the lung group. Therefore, considering blood indicators are more intrinsic, we assumed that the immune activation effect was equal in bone and lung. Based on the analyses, we hypothesized that there were statistical differences in irradiated sites in the activation of immune system and short-term survival during immunotherapy in advanced NSCLC. However, due to the limitation of this retrospective study, the mode, dose, and targeted area of brain RT are also the direction for future studies.

Studies have revealed that primary metastases had various levels of genomic heterogeneity to distinct tumor migration patterns (35). Related genes were enriched in different metastatic locations, which would alter tumor microenvironment and related inflammatory cell distribution and finally influence the response to ICIs (28, 29). The presence of liver metastases (LMs) is associated with lower prognosis in immunotherapy compared to metastases in other organs (36). Hepatic tumor is hyper-vascularized and VEGFR is highly expressed on endothelium, which resulted in the accumulation of MDSCs, the decrease of cytotoxic T cells, and the increase of Tregs (37). Similarly, the negative prognosis in response to ICIs was also shown in bone metastases (38). Though the definitive data were absent, ICIs in patients with stable brain metastases represented a better therapeutic option (39). As mentioned before, not only in immunotherapy do the responses of metastases differ, RT for different organs can also make various immune system changes. A prospective study suggested that irradiation to lung and liver induces a decrease in total and cytotoxic NK cells and an increase in activated CD4+ and CD8+ T cells, while these changes were not seen in nonparenchymal sites (31). However, considering tumor heterogeneity, it is found that, in single NSCLC, evidence characterizing the systemic immune response after RT to different metastatic sites is limited. Our study showed significantly different immune responses after RT to primary or metastatic sites during immunotherapy in advanced NSCLC. As a strategy for RT combined with immunotherapy, patients with stable disease should consider brain metastases irradiation to achieve the greatest immune activation effect and short-term efficacy during ICIs, which is followed by bone, lung, and adrenal gland, successively.

In recent years, RT especially SBRT for oligometastases (OMs) made a big progress in NSCLC. The concept of OMs was firstly proposed in 1995 by Hellman and Weichselbaum, of which the consensus was established by the ESTRO and ASTRO committee in 2020 referring to 1–5 metastatic lesions, with a controlled primary tumor being optional and all metastatic sites being safely treatable (40, 41). Many non-randomized studies have shown that SBRT for OMs is safe and effective with a local control rate of about 80% (42). However, the standard optimal dose, fraction, and lesion number of SBRT are still not clear in clinical NSCLC. Conventional views suggest that SBRT to single-site lesion was enough, but there were recent concerns that question whether multisite therapy was superior to single lesion therapy (43). Due to the organs’ own specific heterogenetic tumor-associated antigens, multisite SBRT may broadly enhance the outcome of immunotherapy by facilitating more antigen released (28, 44). Apart from the number of sites to be verified by prospective clinical trials, the localization of SBRT combined with ICIs also remains unclear. The results of this study can provide a reference for patients with oligometastatic advanced NSCLC. As the immune-activated effects of brain, bone, lung, and adrenal radiation declined in sequence, the selection of SBRT to optimal location may bring greater clinical benefit to patients. There were 6 patients subjected to SBRT in our study; 2 from the brain, 1 from the lung, and 1 from the adrenal gland group reached PR, and 1 from the liver group had SD. The CBR reached 87.5% (5/8), and just one patient irradiated at liver had PD. The considerable results showed SBRT to brain and lung seems more beneficial though it was limited by the small number of patients.

Another concept of “Dissociated Response” (DR) was defined as the coexistence of responding and non-responding lesions within the same patient in the evaluation of cancer systemic therapies (33). The incidence of DR was reported to be about 21.5% in solid tumors and approximately 10% in immunotherapy, which occurs more often in advanced NSCLC (IIIB-IV) than in earlier stages (45). As for subsequent therapy for DR patients, continuing the initial ICIs instead of switching to the next-line antitumor therapy indicated a better survival (46, 47). However, is it necessary to select the locoregional RT site(s) among multiple metastases to achieve more clinical benefits when continuing ICIs? This knowledge may be crucial to refine ongoing and future clinical studies combining RT and immunotherapy in advanced NSCLC, especially in patients with DR.

By means of this clinical retrospective study, we can further explore the mechanism of this phenomenon performed as an immune activation effect of different irradiated sites during ICIs. Understanding the differences of related molecular pathways, inflammatory cell infiltration, and protein expression plays a guiding role in discovering irradiated organs’ heterogeneity, which could maximize the benefit of radioimmunotherapy. In future prospective research, attention should be focused on the following points: firstly, the choice of time point, the best choice of patients treated firstly in order to reduce interference with the results; secondly, the screening of clinical indicators and laboratory molecular phenotypes; and finally, the proper statistical analysis of results.

To date, this is the first study in advanced NSCLC to evaluate the differences between immune system activation and short-term efficacy of radiotherapy for primary lesion or distant metastases during immunotherapy. However, there are several limitations in this study. As it is a retrospective analysis, the characteristics of clinical cases were complex. For example, earlier multi-line treatments and local radiotherapy were involved, which might have delayed the effect on immunotherapy subsequently. Also, there are various kinds of ICIs and regimens, which may influence the efficacy of immunotherapy. Prospective clinical trials are warranted to provide more convincing evidence and specific standards for combination therapy with RT and immunotherapy in advanced NSCLC.

## Conclusion

We have seen the differences in systemic immune activation after RT to the brain, bone, lung, liver, adrenal gland, and soft tissue during immunotherapy synchronously. The lowest medians of delta-MLR and delta-PLR, the highest median of delta-L, and the greatest ORR suggested that irradiation to brain may have the strongest activation effect and best short-term efficacy compared to other organs, which could provide clinical guidance for patients with oligometastases undergoing SBRT and patients with DR. Moreover, we discovered that when the earlier-line ICIs were combined with RT, a better patient outcome was reached. Surely, we could not exclude the fact that the dose, volume, and modes for irradiated sites influenced the observed results. What we hypothesized was that RT to brain compared to other organs may be more immunomodulatory with simultaneous systemic immunotherapy. However, considering the limitations of this study, future prospective studies and lines of evidence are essential for standardizing the specific irradiated metastases, which could maximize the benefits of radioimmunotherapy in advanced NSCLC patients.

## Data Availability Statement

The original contributions presented in the study are included in the article/supplementary material. Further inquiries can be directed to the corresponding authors.

## Ethics Statement

Written informed consent was obtained from the individual(s) for the publication of any potentially identifiable images or data included in this article.

## Author Contributions

MW, JL, SW, JRL, HW, JY, and XM contributed to the study. XM and JY designed the project and approved the final manuscript. JL made critical appraisals. JRL, HW, and SW are responsible for modifying and editing this article. MW collected the clinical patients information and drafted the article. All authors contributed to the article and approved the submitted version.

## Funding

This work was supported by the following grants: National Key Research and Development Projects of China (2018YFC1312201), the Foundation of National Natural Science Foundation of China (81972863, 81627901, and 82030082), National Natural Science Foundation of China (81972864), the Academic Promotion Program of Shandong First Medical University (2019RC002), the Science and Technology Support Plan for Youth Innovation Teams of Universities in Shandong Province (2019KJL001), and the Medical Science and Technology Project of Henan Province (SB201901112).

## Conflict of Interest

The authors declare that the research was conducted in the absence of any commercial or financial relationships that could be construed as a potential conflict of interest.

## Publisher’s Note

All claims expressed in this article are solely those of the authors and do not necessarily represent those of their affiliated organizations, or those of the publisher, the editors and the reviewers. Any product that may be evaluated in this article, or claim that may be made by its manufacturer, is not guaranteed or endorsed by the publisher.
